# Hemangiosarcoma associated with a *Pasteurella multocida* infection in a near threatened cinereous vulture (*Aegypius monachus*): insights into avian pathology and implications for conservation

**DOI:** 10.1186/s12917-025-05220-x

**Published:** 2025-12-23

**Authors:** Sang-Joon Lee, Sangjin Ahn, Taeyeon Kim, Sung-Hyun Moon, Sooyoung Choi, Ho-Seong Cho, Yeonsu Oh

**Affiliations:** 1 College of Veterinary Medicine and Institute of Veterinary Science, Kangwon National University, Chuncheon, 24341 Republic of Korea; 2 Gangwon Wildlife Medical Rescue Center, Chuncheon, 24341 Republic of Korea; 3 College of Veterinary Medicine and Bio-Safety Research Institute, Jeonbuk National University, Iksan, 54596 Republic of Korea

**Keywords:** Aegypius monachus, Avian pathology, Cinereous vulture, Hemangiosarcoma, Pasteurella multocida, Wildlife conservation.

## Abstract

**Background:**

Cinereous vultures (*Aegypius monachus*) are Near Threatened (NT) scavenger species vital to ecosystem health, yet little is known about neoplastic diseases affecting them. Hemangiosarcoma (HSA), a malignant tumor of vascular endothelial origin, is rarely reported in avian species, and its interaction with opportunistic infections remains unexplored.

**Case presentation:**

We report the first case of pulmonary hemangiosarcoma concurrent with *Pasteurella multocida* infection in a cinereous vulture. The adult female bird presented with severe neurological and systemic symptoms, including opisthotonos and emaciation. Diagnostic evaluation involved imaging (CT and MRI), serum chemistry, histopathology, immunohistochemistry, and bacterial culture. Gross pathology revealed a large pulmonary tumor, confirmed as HSA via CD31 immunoreactivity. Concurrent infection with *P. multocida* subsp. septica was identified by culture and 16 S rRNA sequencing, with virulence genes (pfhA, hgbB) detected via multiplex PCR. Neurological signs were attributed to septic encephalopathy.

**Conclusions:**

This case illustrates the complex interplay between neoplasia and bacterial infection in avian species and underscores the importance of multidisciplinary diagnostics in wildlife disease surveillance. Based on the clinical course and pathological findings, the most likely cause of death was acute septicemia with septic encephalopathy secondary to pulmonary hemangiosarcoma and concurrent *Pasteurella multocida* infection. Enhanced awareness of neoplastic conditions in Near Threatened scavenger birds is essential for effective conservation and rehabilitation strategies.

## Background

The cinereous vulture (*Aegypius monachus*) is a large raptor of the Accipitridae family found across temperate Eurasia [1]. Characterized by their impressive size, the males range from 6.8 to 11.8 kg and females from 7.3 to 12.7 kg, with a wingspan of 2.5–2.9 m. These birds are identified by their dark brown plumage, broad wings, short tail, and bluish-gray skin on their heads, necks, and legs. These vultures nest in tall trees across southern Europe and Central Asia, with increasing populations in South Korea [2].

As apex predators, cinereous vultures provide vital ecosystem services by removing carcasses and reducing zoonotic risk. With long lifespans exceeding 20 years in the wild, they serve as valuable sentinel species for environmental monitoring [3]. Despite conservation gains, they remain vulnerable to threats such as poisoning, habitat degradation, and infectious disease. The IUCN Red List classified them as Near Threatened in 2020, highlighting the need for ongoing surveillance [4].

While diseases like avian influenza and botulism are well-recognized, data on non-infectious causes of mortality – particularly neoplasia – are limited [4]. Tumors in wild raptors rarely reported, likely due to diagnostic constraints and possible underrecognition. One case of pulmonary adenocarcinoma in a a lappet-faced vulture remains among the few published examples [4].

Hemangiosarcoma (HSA) is a malignant tumor of endothelial origin, aggressive and prone to hemorrhage. While common in dogs, especially in the spleen and right atrium, it is rarely observed in birds. Avian HSAs are typically cutaneous or coelomic, often presenting with hemorrhagic or necrotic lesions [5]. The pathogenesis remains unclear but may involve inflammation, environmental toxins or immune dysfunction [6].

*Pasteurella multocida*, a Gram-negative, non-motile coccobacillus that serves as an important avian pathogen and possesses recognized zoonotic potential. It is the etiological agent of fowl cholera, predominantly affecting domestic and wild bird species. Importantly, it can also cause opportunistic infections in humans, typically through animal bites, scratches, or close contact with infected birds or mammals. It can cause peracute septicemia through mucosal invasion and systemic dissemination. Its virulence is determined by a variety of factors including capsulr serotype, outer membrane proteins, adhesins (e.g., *pfhA*), and iron acquisition systems (e.g., *hgbB*) [7, 8]. Although *P. multocida* has been isolated from various avian species, including raptors, its co-occurrence with neoplastic disease has not been previously reported.

Here, we report the first case of pulmonary HSA with concurrent *P. multocida* subsp. *septica* infection in a cinereous vulture. This case illustrates the potential interplay between neoplastic and infectious processes in avian wildlife and underscore the importance of combing imaging, histopathology, immunohistochemistry, and molecular diagnostics in wildlife disease investigations. It also reinforces the need for veterinary vigilance in conservation settings and the potential for tumors to impair host immunity and facilitate opportunistic infections.

## Case presentation

**Fig. 1:**
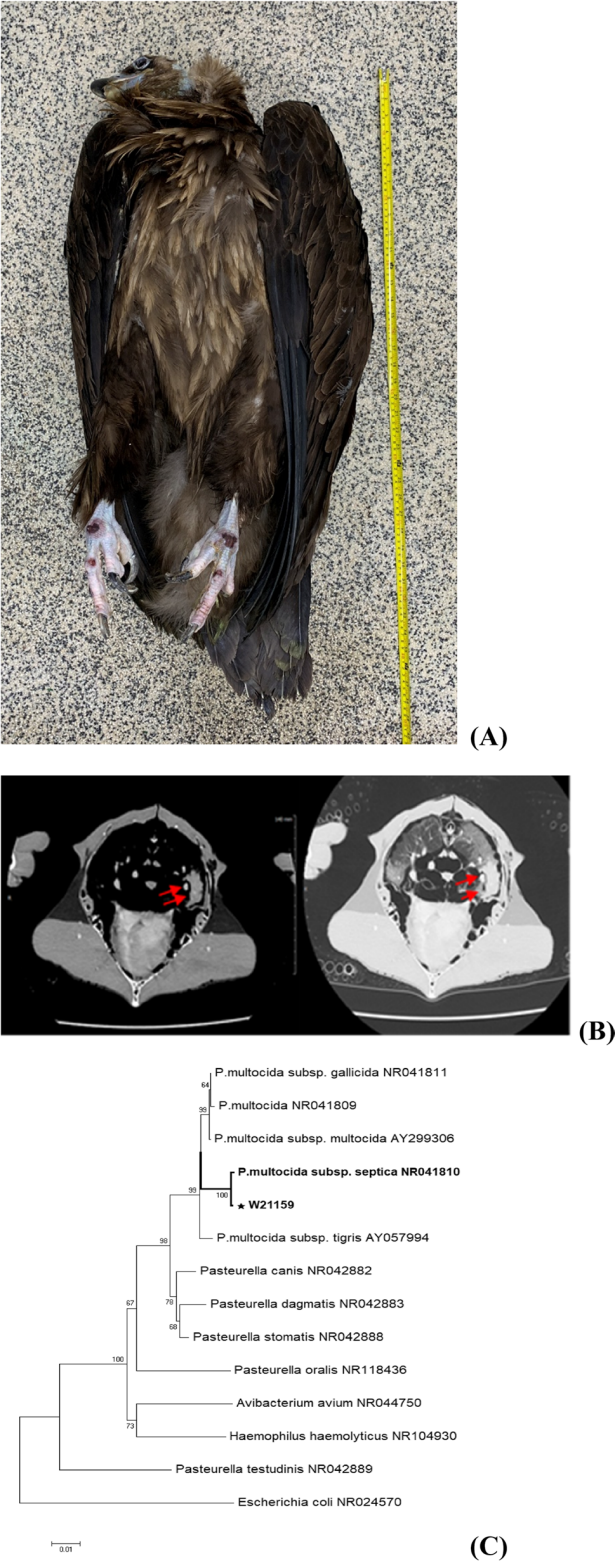
**A** Appearance of the Cinereous vulture (*Aegypius monachus*) on necropsy. **B** Computed tomography (CT) of pectoral area. CT scan result demonstrates there is an abnormal mass in the left side of the lung which is consistent with gross pathology. **C** Phylogenetic relationships of the *P. multocida* based on 16s rRNA genes. The neighbor-joining method phylogenetic tree was calculated by using MEGA11, based on full-length 16s rRNA gene sequences. Numbers at nodes indicate bootstrap values (1000 replicates)

An adult female cinereous vulture (*Aegypius monachus*), previously kept in captivity for approximately four years in a zoo in South Korea, was transferred to the Gangwon Wildlife Medical Rescue Center (WMRC). On arrival, the bird weighed 9.1 kg and showed signs of dehydration, emaciation, lethargy, and inability to stand. A neurological episode characterized by opisthotonos-like head tilting lasted approximately two hours. A visible bruise on the chest and prominent keel indicated severe muscle wasting. A body condition score (BCS) of 1 was assigned using the chicken keel scoring system (Fig. 1A).

**Fig. 2:**
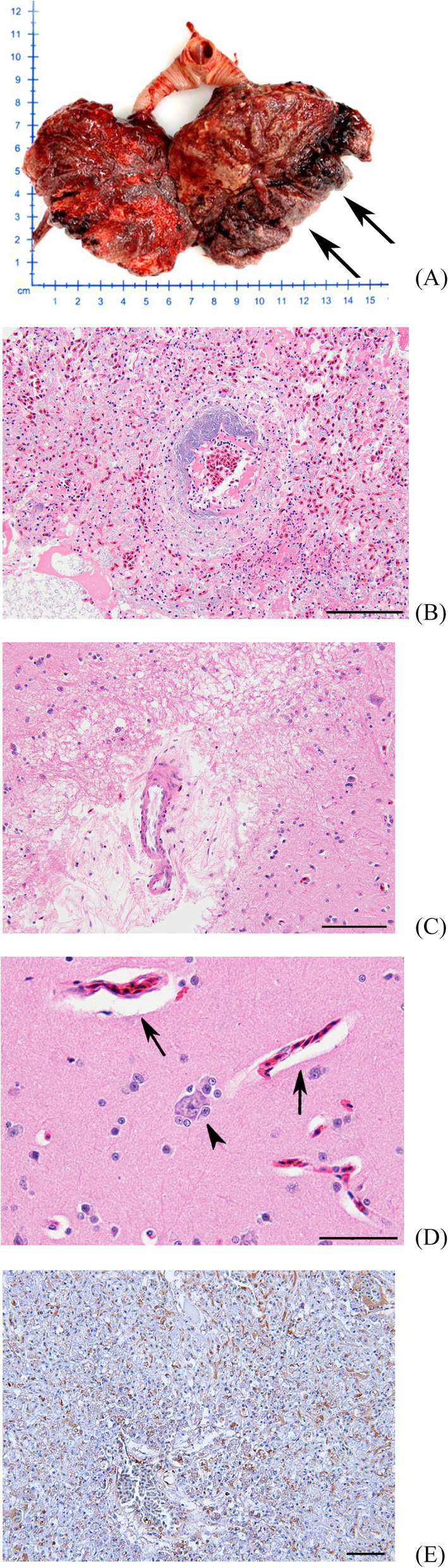
Lung. Cinereous vulture (*Aegypius monachus*). **A** Gross lesion of lung with 8 × 2 × 2 cm tumor mass in the left lobe (arrows). H&E staining, (**B**) Dense and solid pattern of HSA with multifocal bacterial colonies (arrow) presented demarcation from normal lung tissue (upper right). Scale bar = 100 μm, (**C**) Focal demyelination-like tissue loss. Scale bar = 100 μm, (**D**) Congestion with perivascular edema (arrows) and glial satellitosis (arrow head) Scale bar = 50 μm, (**E**) Cytoplasmic immunoreactivity for endothelium with anti-CD31 antibody, DAB chromogen, Mayor’s hematoxylin counterstain, Scale bar = 100 μm

Routine clinical evaluation included a rapid antigen test for avian influenza, which yielded a negative result. Basic hematology and serum chemistry analyses were conducted. The bird exhibited vomiting and diarrhea shortly after transfer to WMRC and succumbed to illness three days later, despite receiving supportive care. The carcass underwent a complete necropsy, during which gross lesions were documented and samples collected for histopathology, bacteriology, and molecular diagnostics. Notable findings included an encapsulated mass measuring approximately 8 cm in the right lung lobe (Fig. 2A).

Taken together with the subsequent bacteriological and histopathological findings, the clinical course was most compatible with acute septicemia and septic shock associated with *P. multocida* infection in the context of an underlying pulmonary hemangiosarcoma.

### Diagnosis and differential diagnosis

Computed tomography (CT) was performed ante-mortem to investigate the pulmonary lesion. The CT scan confirmed the presence of a soft tissue mass within the thoracic cavity (Fig. 1B). Magnetic resonance imaging (MRI) of the brain was also conducted to evaluate potential neurological etiologies for the opisthotonos but did not show significant abnormalities.

Serum chemistry revealed markedly elevated levels of aspartate aminotransferase (AST, 1,028 IU/L; reference range: 184–421 IU/L) and creatine kinase (CK, 6,893 IU/L; reference range: 126-3,000 IU/L), suggestive of extensive muscular and neurological tissue damage. Other parameters, including uric acid, bile acids, glucose, calcium, and protein levels, were within normal limits for avian species.

Detailed hematology values were not available at the time of admission, and thus a complete interpretation of leukocyte response or hydration status could not be performed. Additionally, no significant gross or histological lesions were observed in the liver, kidneys, or other major organs aside from the pulmonary mass and the subtle brain changes described, and this limitation has been noted accordingly.

Histopathological evaluation of the lung mass demonstrated a partially circumscribed neoplasm with obliteration of normal alveolar architecture. The tumor displayed excessive angiogenesis, with vascular channels lined by plump, pleomorphic endothelial cells. Several bacterial colonies were observed in association with neoplastic regions (Fig. 2B). The tumor’s vascular origin was further confirmed via immunohistochemistry (IHC), where endothelial cells showed strong membranous immunoreactivity for CD31, a well-established marker for vascular endothelial tumors (Fig. 2E).

In the brain, histological analysis showed perivascular edema, perineuronal satellitosis (Fig. 2C), and areas of presumed demyelination (Fig. 2D), which correlated with the neurological signs observed during the clinical period.

Postmortem bacterial cultures of lung tissue produced colonies consistent with *P. multocida*. Molecular analysis using 16 S rRNA sequencing confirmed the isolate as *P. multocida* subsp. *septica*. Capsular typing indicated serogroup A, and the isolate was further characterized as sequence type ST334 (RIRDC scheme) and ST33 (multi-host scheme). Multiplex PCR confirmed the presence of two key virulence genes: *hgbB* and *pfhA* (Table 1).

**Table 1:** Epidemiological data of the *P. multocida* isolated from a cinereous Vulture (*Aegypius monachus*) in Korea

Strain	Host	Disease	Capsular serogroup	Sequence type (RIRDC)	Sequence type (multi-host)	Virulence factor			
						toxA	tbpA	hgbB	pfhA
W21159	Cinereous vulture	Pneumonia	A	334	33	-	-	+	+

Differential diagnoses included fungal granulomas, mycobacterial infection, and metastatic neoplasia. However, the combination of histological features, immunoreactivity, and molecular confirmation supported a definitive diagnosis of pulmonary HSA with concurrent *P. multocida* infection.

### Treatment

The vulture received fluid therapy, nutritional supplementation, and supportive care during hospitalization. However, given the severity of clinical signs and rapid disease progression, antimicrobial therapy was not initiated prior to death. This limitation underscores the importance of early diagnosis and intervention in avian medicine, particularly in large, Near Threatened species.

## Discussion

This case represents the first documented occurrence of pulmonary HSA in a cinereous vulture complicated by *P. multocida* infection. Although HSA is well-recognized in dogs, its occurrence in avian species remains exceptionally rare, with only a small number of cases described across various species and anatomical sites [5].

In birds, HSAs have been reported only sporadically across a wide range of species and anatomical locations. Most cases have involved cutaneous or subcutaneous tumors, such as lesions described in a rufous-bellied thrush (*Turdus rufiventris*) (Lima et al., 2016) [6], a Pacific parrotlet (*Forpus coelestis*) (Kline et al., 2016) [7], and blue-fronted and orange-winged Amazon parrots (*Amazona aestiva*, *A. amazonica*) (Gonçalves & Grandi, 2013) [8].

Visceral HSAs are far less common, with examples including an intrathoracic HSA in an ostrich (*Struthio camelus*) (Nakano & Une, 2012) [9] and a metastatic lesion originating from the metatarsal pad of a Java sparrow (*Padda oryzivora*) (Mickley et al., 2009) [10]. Additional earlier descriptions of avian HAS provide useful comparative context (Headley, 2005) [11].

Compared with these predominantly cutaneous or coelomic presentations, the present case of a primary pulmonary HSA in a cinereous vulture represents an uncommon visceral manifestation in a large scavenging raptor and expands the clinicopathological spectrum of avian HSA.

The etiology of avian HSA remains uncertain, with proposed contributing factors including viral oncogenesis, chronic inflammation, and environmental carcinogens. In the present case, no evidence of external lesions or chronic ultraviolet injury was observed. The tumor was confined to pulmonary tissue without metastasis, but histolpathology demonstrated irregular vascular vascular channels and CD31-positive endothelial cells – findings consistent with visceral HSA in mammals [6, 12].

Neoplasia in wildlife is often underdiagnosed due to limited access to advanced diagnostics, rapid disease progression, and the difficulty of detecting internal tumors in free-ranging animals. The highly vascular nature of HSA predisposes to hemorrhage, anemia, and sudden death, which may result in such cases being overlooked without postmortem examination. Here, the combination of CT and MRI imaging, histopathology, immunohistochemistry, and bacterial culture enabled a definitive diagnosis and a detailed understanding of disease pathogenesis.

The architecture and vascular permeability of HAS may have facilitated bacterial colonization. The *P. multocida* subsp. *septica* isolate identified carried *pfhA* and *hgbB*, virulence genes associated with host adhesion and iron acquisition. Iron acquisition systems are particularly important in the nutrient-limited environment of host tissues, and the abdominal vascularture of tumors may provide an accessible niche for bacterial growth [7, 13]. In addition to its significance as an avian pathogen, *P. multocida* has recognized zoonotic potential, with documented cases of opportunistic infection in humans following animal bites, scratches, or contact with infected carcasses. In the context of wildlife rehabilitation, conservation programs, and field research, the presence of this pathogen in a Near Threatened scavenger secies poses a relevant occupational health risk [7, 8, 13, 14].

Immunosuppression secondary to neoplasia may further increase susceptibility to opportunistic infection [9, 15]. In this vulture, vascular remodeling and necrosis within the tumor could have enabled bacterial translocation from the respiratory tract to the bloodstream. Histological detection of bacterial colonies within the tumor supports this interpretation. The combined effect of tumor-associated immune impairment and *P. multocida* septicemia likely contributed to the rapid clinical deterioration observed and ultimately death.

Neurological signs, including opisthotonos, were consistent with systemic effects of bacterial endotoxins and septic encephalopathy, supported by perivascular edema, perineuronal satellitosis, and localized myelin loss in the brain. Elevated serum CK and AST supported the presence of widespread neuromuscular damage [4].

From a conservation persepective, this case underscores the importance of considering neoplasia alongside infectious disease when evaluating mortality in threatened avian species. Standardized postmortem protocols, tissue archiving, and integration of advanced imaging and molecular diagnostics into wildlife rehabilitation settings could improve detection rates for both tumor and pathogen-related mortality. Importantly, the zoonotic potential of *P. multocida* emphasized the need for biosecurity protocols and personal protective euipment for personnel handling with birds, particularly in multispecies rehabilitation facilities.

## Conclusion

This case highlights the complex interplay between neoplastic and infectious disease processes in a Near Threatened avian scavenger. Pulmonary HSA in the cinereous vulture likely created a permissive environment for *P. multocida* infection, with tumor-induced immunosuppression and vascular pathology facilitating bacterial colonization. The findings demonstrate the value of a multidisciplinary diagnostic approach – combining advanced imaging, histopathology, immunohistochemistry, and molecular pathogen – for comprehensive wildlife disease investigation. Beyond veterinary relevance, the zoonotic nature of *P. multocida* underscores the One Health importance of such cases, informing not only conservation medicine but also occupational health and public health surveillance.

## Data Availability

Not applicable.
